# Biomedical Use of Isothermal Microcalorimeters

**DOI:** 10.3390/s101009369

**Published:** 2010-10-18

**Authors:** Olivier Braissant, Dieter Wirz, Beat Göpfert, A.U. Daniels

**Affiliations:** Laboratory of Biomechanics & Biocalorimetry, Biozentrum/Pharmazentrum, University of Basel, Klingelbergstrasse 50-70, 4056 Basel, Switzerland; E-Mails: dieter.wirz@unibas.ch (D.W.); beat.goefpert@unibas.ch (B.G.); au.daniels@unibas.ch (A.U.D.)

**Keywords:** microcalorimeters, bacteria, human cell lines, antimicrobial agent testing, materials biocompatibility

## Abstract

Isothermal microcalorimetry is becoming widely used for monitoring biological activities *in vitro*. Microcalorimeters are now able to measure heat production rates of less than a microwatt. As a result, metabolism and growth of relatively small numbers of cultured bacteria, protozoans, human cells and even small animals can be monitored continuously and extremely accurately at any chosen temperature. Dynamic effects on these organisms of changes in the culture environment—or of additions to it—are easily assessed over periods from hours to days. In addition microcalorimetry is a non-destructive method that does not require much sample preparation. It is also completely passive and thus allows subsequent evaluations of any kind on the undisturbed sample. In this review, we present a basic description of current microcalorimetry instruments and an overview of their use for various biomedical applications. These include detecting infections, evaluating effects of pharmaceutical or antimicrobial agents on cells, monitoring growth of cells harvested for tissue eingineering, and assessing medical and surgical device material physico-chemical stability and cellular biocompatibility.

## 1. General Introduction to Isothermal Microcalorimetry (IMC)

Physiological studies using isothermal microcalorimetry are not new. During the 18th century scientists used an “ice calorimeter” to monitor heat release by small animals [1,2]. Since these first studies several types of calorimeters have been developed and have of course become much more sensitive and accurate. The different types of calorimeters can mainly be distinguished based on the fate of the heat produced or consumed in the calorimeter and the thermal and physical relation of the calorimeter to its surroundings (see [3] for a review). Among the most common types are differential scanning calorimeters, titration calorimeters and accelerated rate calorimeters. In this review the focus is on another increasingly common type—isothermal microcalorimeters.

Isothermal microcalorimeters (IMCs) are defined as instruments measuring heat flow in the microwatt range (μW = μJ/s) and operating at nearly constant temperature [4]. In this type of calorimeter the heat produced or consumed in the calorimetric ampoule is allowed to flow between the ampoule and a heat sink (usually an aluminum block) thus keeping the calorimetric ampoule and its contents within a few millidegrees of the temperature at which the heat sink is maintained by the thermostatic system in which the calorimeter operates. The real sensing elements are the thermoelectric modules (*i.e.*, Seebeck or Peltier modules) placed between the sample and the heat-sink. These thermoelectric modules allow any slight temperature difference to be converted into an electrical signal, which can be easily recorded (see [3] for an instrument design review).

Over the last 30 years, the growing availability of multi-channel isothermal calorimeters and microcalorimeters has made rapid, multi-variable biological studies with requisite replicate specimens practical in various fields. These include soil and environmental sciences [5–7], medical and environmental microbiology [8,9], and food science [10]. In addition to the increasing availability of conventional calorimetry instruments, different “microcalorimeters on a chip” have been developed and described within recent decades as well [11]. Both “chip” and conventional microcalorimeters are of great value for biomedical applications and research.

The purposes of this review are to describe the many uses of microcalorimetry that have been made in biomedical sciences using conventional or chip calorimeters, and to give the reader an overview of the possibilities offered by each type of instrument. This review does not include isothermal titration calorimetry (ITC) techniques and applications. ITC is used extensively for studying ligand binding interactions, and ITC deserves a separate review. However several IMC-related uses of ITC will be presented in the application examples.

## 2. Main Advantages and Drawbacks of Isothermal Microcalorimetry (IMC)

IMC presents an interesting set of advantages [8,12]. IMC allows dynamic measurements of virtually any chemical or physical process by measuring related heat production or consumption. It can accomplish this in small specimens (e.g., g or mL range and smaller) because it can measure very small heat production rates by measuring extremely small temperature differences. This makes IMC not only a sensitive method able to measure heat flow on the order of nano or microwatts but also provides a passive assessment of chemical and physical processes since only heat is measured. IMC thus neither requires the sample to be modified in any way (e.g., with an added fluorescent or radioactive labels) nor does it alter the sample during measurement. Therefore the samples do not require a specific preparation for IMC, and they can still be subjected to further measurements after the microcalorimetric measurement. In addition, rate processes involving samples that are difficult to monitor in real time using other methods—e.g., ones taking place on opaque solids or in porous samples—can be monitored easily using microcalorimetry.

On the other hand the chief drawback of microcalorimeters should be mentioned. This is that isothermal microcalorimeters (as other types of calorimeters) measure a non-specific signal. The net heat flow of all processes releasing or consuming heat in an IMC ampoule are recorded. This emphasizes the importance of careful experimental planning, e.g., also recording heat flow for ampoules containing only the specimen environment and no specimen, and making post-IMC assessments of the specimen and the specimen environment to ascertain what changes have been measured by recording heat flow.

## 3. Conventional Isothermal Microcalorimeters *vs.* Chip Microcalorimeters

One of the main differences between large-ampoule microcalorimetric instruments and chip microcalorimeters is the amount of sample required. Large ampoule instruments are designed to accept microcalorimetric ampoules with volumes between 1 and 20 mL and will usually only accept ampoules of a given size (or similar size), while chip microcalorimeters can have reaction chambers with volumes as low as 0.7 nL [13]. These very low volumes can be of great value if expensive or only miniscule amounts of biological compounds or implant materials are available for evaluation. In addition, the small chamber size of a chip microcalorimeter results in a much smaller time constant (between 10 ms and a few seconds—[11,13]) compared to larger instruments (e.g., up to a few minutes seconds for a 20 mL ampoule—[14]). Similarly in chip microcalorimeters, equilibration time is much shorter due to the extremely small sample volume that must be brought to the set temperature (on the order of seconds to minutes). In material testing the very small time constant of chip microcalorimeters make them usable not only as an IMC instrument but also as a differential scanning calorimeter (DSC). They can therefore determine important material thermal properties such as glass transition temperatures [15,16]. However chip microcalorimeters trade sample volume for sensitivity [11,17]. The specifications for sensitivity and volume-specific heat power resolution for chip and conventional microcalorimeters are summarized in Table 1. Because of these trade-offs, conventional isothermal microcalorimeters are still often preferred to chip microcalorimeters and are the instruments currently in widest use.

Both conventional IMCs and chip microcalorimeters can be calibrated using various techniques. The most commonly used calibration method in both cases is an integrated electric heater producing a known thermal power [18,19]. In addition several chemical reactions such as acid-base reactions, hydrolysis of sucrose, and phase changes such as dissolution or melting of solid compounds can be used to calibrate microcalorimeters (see [19] for a review). For chip microcalorimeters a laser of known power can be used as well, as described in [18].

## 4. Biomedical Applications of IMC

The use of a label-free, passive, dynamic measurement (*i.e.*, heat flow) opens a wide range of potential use in biomedical sciences. In terms of biomedical and clinical applications it allows investigating the metabolism and growth of human cell cultures and also potentially infectious, contaminated or genetically modified organisms in an environment of choice. Of equal importance, it is often possible to use closed ampoules and still obtain reliable measurements of activity and growth—thus under safe and controlled conditions. In addition, many processes relevant to biomaterial degradation or material stability can be studied using IMC. Several fields of application are described below with specific examples in each case.

### 4.1. Detection of Infections

Calorimetry has been extensively used to monitor microbial activies [4,8], most probably because of the fast-growing nature of microorganisms and the resultant exuberant amount of heat that is soon produced. In the medical field it has been shown that microcalorimetry can be an excellent tool to rapidly detect infection or microorganism contamination of clinical products or samples. Rapid detection of the contamination of platelets by different bacteria was achieved within a few hours [20]. In addition it was shown that rapid detection of methycillin-resistant *Staphylococcus aureus* could also be achieved within a few hours [21,22]. Similarly isothermal microcalorimetry has proven to be highly effective in detecting slow-growing bacteria as rapidly as possible. For example it was shown that mycobacteria and especially *Mycobacterium tuberculosis* could be detected within hours to a few days using microcalorimetry [23]. This is much faster than traditional culture methods that can take up to 60 days to detect growth of slow growing mycobacteria. The use of a chip microcalorimeter was also investigated to detect pathogens. Growth of *Escherichia coli* was successfully monitored [24]. In addition, more specific detection was achieved with a chip microcalorimeter using the reaction between *Neisseria meningitides* and its specific antibody HmenB3 [25]. The results of this last study were comparable to ELISA testing. It should be noted that gene-based methods for detecting microorganisms (and also their resistance strains) can be quite rapid. However, several gene-based methods do not indicate whether or not the microorganism is a live one (replicating or at least metabolically active), and gene-based identification of resistance is limited to microorganisms for which resistance-related genes have already been identified.

### 4.2. Antibiotic Testing

Microcalorimetry has been used extensively since the 1980s to perform antibiotic bioassays [26–30] using various types of microcalorimeters. More recently a comparison between traditional and microcalorimetric determination of minimal inhibitory concentration (MIC) for several antibiotics was performed [30]. This study concluded that MICs determined using microcalorimetry were virtually the same as those determined according to CLSI (Clinical Laboratory & Standards Institute) recommendations. But IMC had the additional advantage at sub-inhibitory concentrations of easily showing the mode of action of the various antibiotics (*i.e.*, bacteriostatic *vs.* bacteriocidal). Many potential new antibacterial compounds and antibacterial coatings for implant materials are being tested now using microcalorimetry [31–36] and similarly several antivirals have also been tested [37]. In addition to antibiotic bioassays and MIC determinations, microcalorimetry is an excellent possible tool for monitoring in real time the effects of antibiotics on biofilm formation *in situ* [38]. However, except for this example, few IMC studies have focused to date on the effects of antibacterial compounds on biofilm activity.

**Figure 1.**
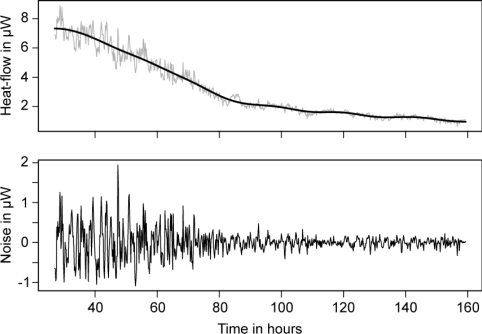
Microcalorimetric study of *Schistosoma mansonii* metabolic heat with addition of an anti-parasitic compound. **Upper panel:** Raw thermogram (grey line) and thermogram smoothed using wavelet smoothing (black line). **Lower panel:** Noise calculated by subtracting the smoothed thermogram from the raw thermogram. Note the decreases in the noise amplitude, down to a constant minimum level. This decrease corresponds to the decrease in worm motor activity. Figure modified from [39] with permission from Elsevier.

Finally it must be noted that IMC has recently been recognized as a tool able to accurately determine efficiency of an anti-parasite drug on *Schistosoma mansonii* adult worms [39]. This IMC method is based on determining drug-induced changes in both the organism’s metabolic heat production and motor activity (Figure 1). IMC not only continuously quantifies the effects of drugs on worm viability by recording overall metabolic heat output, but also quantifies metabolic fluctuations produced as a result of the worm moving or flexing its body.

### 4.3. Implant Material and Other Biomedical Material Testing

As mentioned above, one of the main advantages of microcalorimetry is that it can accommodate any type of sample. For implant and other biomedical material testing this allows introducing a solid sample into a microcalorimeter ampoule. One can then continuously record heat flow (relative to controls) and estimate the rate and extent of processes such as degradation (e.g., hydrolysis, corrosion), and biologic phenomena such as adhesion of cells (e.g., bacteria) to material surfaces.

**Figure 2.**
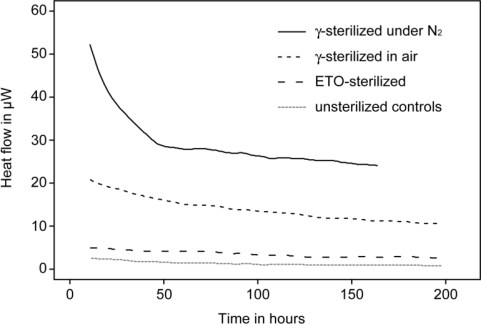
Microcalorimetric study of UHMWPE sterilization. Gamma irradiated samples had a much higher activity compared to ethylene-oxide sterilized samples and unsterilized controls. Figure modified from [42].

Among polymeric implant materials polyethylenes (PE) are among the most important. Ultra-high molecular weight polyethylene (UHMWPE) in particular is widely used as one of the bearing surfaces in hip, knee and other joint replacement implant designs. The type of pre-implantation sterilization process used on UHMWPE has a strong influence on the slow (months, years) but steady oxidative processes which affect the material’s structure and mechanical properties. Specifically gamma irradiation creates free radicals in UHMWPE, and unless this is thwarted by some means (e.g., addition of α-tocopherol) it increases oxidation process rates. The oxidation of UHMWPE leads to embrittlement, compromising mechanical integrity (e.g., wear resistance, fracture resistance) and thus clinical performance in joint replacement applications [40]. In this context several microcalorimetric studies have evaluated stability of UHMWPE. One of these studies demonstrated that physico-chemical stability of gamma sterilized UHMWPE implant material was five to nine times less than the ethylene oxide (EtO) sterilized counterpart (Figure 2) [41,42]. The authors hypothesized that this lower stability was due to a combination of free radical reactions and hydrolytic degradation of the oxidized part of the polymer. In a later study, similar results were found in a comparison of heat production rate of gamma sterilized, EtO sterilized or gas plasma sterilized UHMWPE slices. In addition the authors tested the influence of atmosphere at the time of gamma irradiation and subsequent storage, and concluded that there was no difference between samples irradiated under N_2_ atmosphere or sample irradiated under air atmosphere if the time of exposure to air (*i.e.*, oxygen) prior to IMC evaluation was accounted for [42].

Similarly, bone cements have been extensively studied by isothermal microcalorimetry. For calcium phosphate based cements, setting/curing time of cements are of great importance since too fast setting/curing will lead to lower strength and toughness [43]. Like many other solid-state reactions, cement hydration cannot be described by ordinary chemical rate equations (first order, second order, *etc.* [44]). However typical stages in cement setting can be distinguished. First rapid initial processes (usually not seen during IMC measurements due to thermal equilibration time required after inserting an ampoule containing a cement that is in the process of setting), then a lag period, then a rapidly increasing reaction rate after initialization, and finally a decreased reaction rate [14,44]. Monitoring setting using IMC makes it possible to follow the evolution over months if desired. (Specimens in sealed ampoules can be maintained outside the calorimeter at the measurement temperature, and then inserted periodically into the calorimeter to measure the current heat flow rate.) Therefore isothermal microcalorimetry is an excellent tool to study the effect on setting reactions of candidate additives used to improve strength or other properties of such bone cements; alternatively differential scanning calorimeters can and have been used in isothermal mode to perform such measurements. Also, using this approach, influence of particle size or milling time on the setting reaction kinetics of calcium phosphate based cements were successfully studied [45,46]. Similarly the effect of citric acid as a retardant on the setting time of brushite-forming cement was studied by Hofmann *et al.* [43]. These authors emphasized that the setting time effects on mechanical properties measured by indentation methods were strongly correlated with the maximum heat flow measured with a DSC instrument used in isothermal mode. However these authors also noted several drawbacks of their calorimetric approach. The first minutes of the reaction were lost due to the preparation of sample outside the calorimeter and time needed in the calorimeter to first achieve thermal equilibration. In addition they also mention that only exothermic reactions could be studied. However others have found that true IMC instruments allow early-stage measurements using either home-made injection systems or commercially available systems such as Admix^®^ ampoules (for the TAM Air^®^ and TAM III^®^ calorimeters).

For acrylic-based bone cements, studies have shown that setting time measurements are also possible with IMC [47]. In addition some other IMC studies suggest that in the case of acrylic cements the sterilization method has important effects on cement stability [48] for reasons similar to those described above for UHMWPE implant material.

Finally for all common types of implant materials (ceramics, polymers, metals) isothermal microcalorimetry allows estimating the rate and extent of bacterial adhesion on surfaces. IMC is especially useful for comparing the effects of different materials or material surface treatments. Several studies have used isothermal titration calorimetry to approach this issue [49,50]. However a simpler method was used by Hausser-Gerspach *et al*. [51] to show that IMC could differentiate effects of medium (saline *vs.* saliva) and material surface area, using a glass bead model. Also, injection systems like those described above can be used to capture the initial stages of adhesion processes. Studies under way in the authors’ laboratory suggest that IMC can be used in analogous fashion to study adhesion of mammalian cells on materials.

### 4.4. Microcalorimetry of Engineered Tissues, Tissue Samples and Biopsies

**Figure 3.**
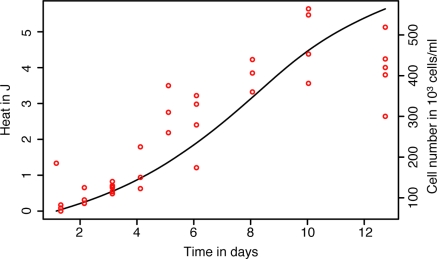
Monitoring chondrocyte growth in 3 mL microcalorimetric ampoules. The line represents the total heat produced, and the dots represents the cell counts.

Engineered tissues have been the focus of intensive research and development for several years for potential use in surgical repair. In connection with tissue engineering, microcalorimetry has been shown to be interesting as a means for following the expansion (growth) of human cell lines under various culture conditions. Studies performed with CHO320 hamster ovarian cells have emphasized that microcalorimetry could be used for media optimization [52]. However microcalorimetry need not be restricted to use in optimization. It has been shown that growth of primary human chondrocytes could easily be followed in a microcalorimetric ampoule and that initial IMC-measured growth rates reflect growth during longer periods under more standard expansion conditions as determined by cell counts—a highly time-consuming procedure. Thus IMC may be a simple alternative means for assessing the expansion potential of cells from a given donor. In addition microcalorimetry allows following real-time growth of chondrocytes in candidate tissue engineering scaffold materials (R. Santoro, I. Martin personal observation—Figure 3) without interference and without using destructive methods to monitor growth. Such an approach has also successfully been used to monitor the activity of microencapsulated cells [53]. Since microcalorimetry is a non-destructive method, high-value samples can be recovered intact for further analyses. In the case of chondrocytes in scaffolds this allows post-IMC evauation of the biochemistry, histology and mechanical properties of the nascent cartilage [54,55].

Similarly monitoring of dissected living tissues or living tissues from biopsies has proven to be of great value for research and diagnostic purposes (see [56,57] for a review). Early calorimetric studies on muscles where performed with a thermopile directly inserted in the tissues [58,59]. “Home-made” thermopiles have been created and used to understand the energy balance during frog Sartorius muscle contraction [60]. Later studies combined this approach with other biochemical measurements to get more insight into the biochemichal reactions responsible for the generation of heat [61].

**Figure 4.**
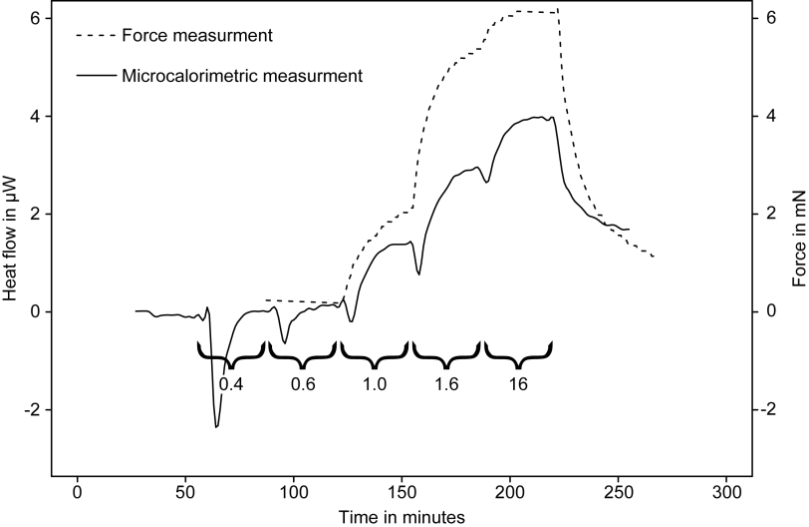
Parallel monitoring of the heat production (plain line) rate and force generated (dashed line) by chemically skinned smoothed muscle upon addition of calcium chloride. Numbers under backets indicate the calcium concentration added in μM. Figure modified from [62] with permission of John Wiley & Sons Ltd.

Subsequent studies used conventional microcalorimeters to study heat production of guinea pig taenia coli muscles under various conditions. In this case the muscle was attached to a modified stirrer that was placed in a 4 mL stainless steel ampoule containing 2.7 mL of relaxant solution. The muscle contractions were induced by adding calcium chloride into the solution using the perfusion or titration unit to which the stirrer was attached [62]. In their synchronized parallel experiment the authors showed that the muscle contraction and work measured using a force transducer were in perfect agreement with heat production rate (heat-flow) measured by the microcalorimeter (Figure 4).

Similar to the dissected animal muscles studies, microcalorimetry has been used to investigate the effects of drugs on human muscle biopsies. Effects of propanolol and other β-blockers have been of particular interest. It was shown that muscle biopsied from a patient who had received propanolol for one week had a lower thermogenesis as well as a lower isokinetic endurance [63]. Similarly later studies obtained analogous results in human and animal models and emphasized the differences between propanolol and other drugs such as terbutaline [64] and carvedilol [65].

Finally, IMC performed on urogenital tract biopsies was shown to be effective in discriminating tumorous from non-tumorous tissues [66]. In addition, through comparison of histological investigations and calorimetry data these authors emphasized that it was possible to grade the malignancy of the tumor using the heat production data.

**Figure 5.**
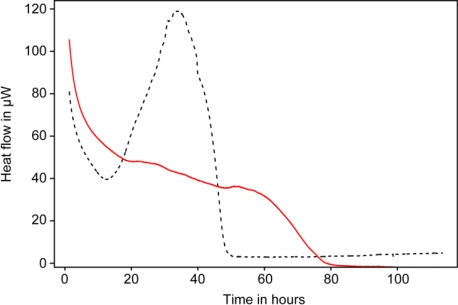
Example of calorimetric monitoring of biopsies. The plain line shows the heat production decrease of a bone tumor sample over time. The dashed line (from another sample) shows an extraneous peak due to an infection by bacteria.

Until now this study is the only one to the authors’ knowledge that has applied and validated such use of isothermal microcalorimetry. However preliminary data obtained in the authors’ lab show that heat production from tumorous bone biopsies is indeed easily detected using calorimetry. In addition as mentioned earlier, superinfection of bone tissue is easily detected as well (Figure 5).

## 5. Concluding Remarks

Calorimetry and microcalorimetry have been used for a long time in basic and applied studies in the general fields of reaction rate chemistry and physical chemistry. However, as demonstrated above, the last decade has seen many new biologic applications arise. This is in great part a consequence of the development and availability of very sensitive and multichannel instruments. In addition concomitant advances in computer processor performance and data storage have facilitated rapid and more detailed analysis of large amounts of IMC data. The recent work reviewed here makes it seem likely to the authors that within the next few years clinical usage will become more common and many more research studies focusing on important biomedical questions will be performed. These studies will complement the early studies performed on human and aminal cells (see [27] for a review). Although microcalorimetry is a “universal and versatile” detection method, it also seems likely to the authors that many of these studies will focus on such topics as thermodynamics and kinetics at the cell-material interface and cell growth in porous implant materials, tissue engineering scaffolds and in other biotechnological applications. For example the passivity of IMC measurements would make it an excellent tool for monitoring growth and/or contamination of engineered tissues without interference or sampling of such precious materials, and thus help ensure the quality of the process and the product. In addition calorimetry, in general, is a fast growing field. Many new instrument designs are emerging [67–69] and in the biosciences may lead to high-throughput calorimetric screening and testing of genetically modified cells, pharmaceuticals, and medical biomaterials.

## Figures and Tables

**Table 1.** Specifications of several chip microcalorimeters and available conventional IMC instruments. Note the higher specific sensitivity of the conventional instruments.

**Microcalorimeter type**	**Volume [mL]**	**Detection limit [μW]**	**Specific sensitivity [mW × L^−1^]**	**Reference**
**Chip calorimeters**				

Johannessen *et al.*	7.2 × 10^−7^	1.3 × 10^−2^	1.8 × 10^4^	[13]
Zhang *et al.*	1.5 × 10^−5^	5 × 10^−2^	3.3 × 10^3^	[69]
Torres *et al.*	5.0 × 10^−4^	1 × 10^−1^	2.0 × 10^2^	[67]
Lerchner *et al.*	5.0 × 10^−3^	5 × 10^−2^	1.0 × 10^1^	[17]
Higuera-Guisset et al.*	6.0 × 10^−1^	1 × 10^−1^	1.7 × 10^−1^	[24]

**Conventional calorimeters**				

Calmetrix I-Cal 8000^®^	1.25 × 10^2^	2 × 10^1^	1.6 × 10^−1^	†
Waters/TA TAMair^®^	2 × 10^1^	2.5	1.3 × 10^−1^	†
THT μMC^®^	1.5–4.0	2 × 10^−1^	1.3 × 10^−1^–5.0 × 10^−2^	†
Waters/TA TAM48^®^	1.0–4.0	2 × 10^−1^	2.0 × 10^−1^–5.0 × 10^−2^	†
Waters/TA TAM III^®^**	1.0–4.0	2 × 10^−2^	2.0 × 10^−2^–5.0 × 10^−3^	†
Setaram C80^®^	1.25 × 10^1^	1 × 10^−1^	8.0 × 10^−3^	†

* Indicates that the microcalorimeter is based on a Xensor^®^ chip microcalorimeter;

** Waters/TA differential nanocalorimeter;

† manufacturer’s data.
